# Mobile microscopy as a screening tool for oral cancer in India: A pilot study

**DOI:** 10.1371/journal.pone.0188440

**Published:** 2017-11-27

**Authors:** Arunan Skandarajah, Sumsum P. Sunny, Praveen Gurpur, Clay D. Reber, Michael V. D’Ambrosio, Nisheena Raghavan, Bonney Lee James, Ravindra D. Ramanjinappa, Amritha Suresh, Uma Kandasarma, Praveen Birur, Vinay V. Kumar, Honorius-Cezar Galmeanu, Alexandru Mihail Itu, Mihai Modiga-Arsu, Saskia Rausch, Maria Sramek, Manohar Kollegal, Gianluca Paladini, Moni Kuriakose, Lance Ladic, Felix Koch, Daniel Fletcher

**Affiliations:** 1 Department of Bioengineering, University of California, Berkeley, CA, United States of America; 2 Head and Neck Oncology, Mazumdar Shaw Medical Centre, NH Health city, Bangalore, India; 3 Integrated Head and Neck Oncology Program (DSRG-5), Mazumdar Shaw Center for Translational Research, Mazumdar Shaw Medical Foundation, NH Health City, Bangalore, India; 4 Siemens Healthcare Pvt Ltd, Bangalore, India; 5 Department of Pathology, Mazumdar Shaw Medical Centre, NH Health City, Bangalore, India; 6 Department of Oral and Maxillofacial Pathology, KLE Society’s Institute of Dental Sciences, Bangalore, India; 7 Department of oral medicine and radiology, KLE Society’s Institute of Dental Sciences, Bangalore, India; 8 Siemens S.R.L, Brasov, Romania; 9 Siemens Healthcare GmbH, Erlangen, Germany; 10 Siemens Medical Solutions USA Inc., Princeton, NJ, United States of America; 11 Department of Oral and Maxillofacial Surgery–Plastic Surgery, University of Mainz, Mainz, Germany; Emory University/Georgia Institute of Technology, UNITED STATES

## Abstract

Oral cancer is the most common type of cancer among men in India and other countries in South Asia. Late diagnosis contributes significantly to this mortality, highlighting the need for effective and specific point-of-care diagnostic tools. The same regions with high prevalence of oral cancer have seen extensive growth in mobile phone infrastructure, which enables widespread access to telemedicine services. In this work, we describe the evaluation of an automated tablet-based mobile microscope as an adjunct for telemedicine-based oral cancer screening in India. Brush biopsy, a minimally invasive sampling technique was combined with a simplified staining protocol and a tablet-based mobile microscope to facilitate local collection of digital images and remote evaluation of the images by clinicians. The tablet-based mobile microscope (CellScope device) combines an iPad Mini with collection optics, LED illumination and Bluetooth-controlled motors to scan a slide specimen and capture high-resolution images of stained brush biopsy samples. Researchers at the Mazumdar Shaw Medical Foundation (MSMF) in Bangalore, India used the instrument to collect and send randomly selected images of each slide for telepathology review. Evaluation of the concordance between gold standard histology, conventional microscopy cytology, and remote pathologist review of the images was performed as part of a pilot study of mobile microscopy as a screening tool for oral cancer. Results indicated that the instrument successfully collected images of sufficient quality to enable remote diagnoses that show concordance with existing techniques. Further studies will evaluate the effectiveness of oral cancer screening with mobile microscopy by minimally trained technicians in low-resource settings.

## Introduction

Oral cancer, through a convergence of behavioral and infrastructural factors, is the most common type of cancer among men in India and other countries of South Asia [1, 2]. Tobacco and alcohol-related habits predispose individuals to the development of both precancerous lesions and oral cancer [3], and the higher proportion of men with these habits is thought to contribute to the gender differences in oral cancer incidence [2]. Additionally, data from case-control and meta-analytic studies have shown that HPV is also an independent risk factor for the development of oropharyngeal and oral carcinomas [4, 5]. The stage of disease at presentation is a crucial prognostic factor for oral squamous cell carcinoma [6, 7]. While the 5-year survival rate of oral cancer diagnosed at stage I and II is over 80%, in stages III or IV it is less than 40% [6], emphasizing the need for earlier diagnosis of lesions.

Definitive diagnosis of oral cancer is achieved by performing biopsy followed by histological examination of suspicious lesions [8–10]. This method involves examination of the oral cavity and the use of either a scalpel or a punch biopsy and histopathologic processing followed by a specialist’s interpretation. The sample collection is invasive and painful, however, improper sample handling and transfer to a histology facility can preclude its use for providing a diagnosis [11]. As a result, biopsies are normally performed in a hospital setting or under the direction of a skilled clinician. The first point of contact for the majority of patients seeking care, particularly in a rural setting, is not a centralized facility that can provide these types of services. A visual examination for suspicious lesions is the standard screening approach for referring patients from a primary care setting to a centralized location for biopsy and care [7, 12, 13], but its specificity for precancerous lesions is low. This in turn reduces the likelihood of a care provider directing a patient for further care and reduces compliance of the patient for a painful secondary procedure. In many South Asian countries, this is further compounded with tobacco and betel quid usage, leading to a large number of nonspecific lesions in screened patients. This scenario would benefit significantly from a more sensitive and specific screening tool that is appropriate for the infrastructure and personnel at peripheral clinics [7].

Many technologies have been developed over the past several decades in an attempt to improve examination of the oral cavity when screening or potentially diagnosing oral pre-cancer or cancer [7, 14, 15]. Several of these are aids to visual inspection of the oral cavity, effectively increasing the contrast between dysplastic or malignant tissue and normal tissue. These include toluidine blue staining [16] for detecting the increased nuclear content in malignant lesions and use of acetic acid with blue light to produce chemiluminescence, which can potentially improve visualization of oral lesions [17]. While these are potentially simple ways to improve the standard visual screening, studies report poor specificity of these techniques and moreover they are not validated with gold standard biopsy data [7]. Other potential techniques include autofluorescence imaging of metabolic intermediates that indicate cancer phenotypes, broad-spectrum reflectance and scattering that can probe architecture [18] and optical coherence tomography (OCT) [19]. While promising, these techniques have had limited acceptance and require custom instrumentation that is often unavailable at the point of care.

A screening tool that has been successfully evaluated across a range of medical setting is brush biopsy [20, 21], which involves minimally invasive sampling of the suspicious region with a brush followed by slide preparation and pathologist interpretation with a transmission light microscope. Cytological information gathered from these samples can indicate cellular changes including nucleus-to-cytoplasm ratio and nuclear structure. This technique can be a useful aid for differentiating benign lesions from potentially cancerous lesions, enabling a frontline health worker to make a more effective referral to a central facility for final diagnosis. However, this technique has not been available in peripheral clinics due to challenges associated with the need for microscopes and trained personnel to interpret the images.

The proliferation of mobile devices and the supporting data transfer network has the potential to leapfrog the need for establishing traditional infrastructure in the peripheral locations where care is administered. These consumer electronic devices deliver an intuitive user interface, significant computational power, and high-quality imaging optics in a low-cost package, while the ability to transfer data can bring expertise to remote locations with limited overhead. A range of devices has been developed to speed traditional diagnostics or enable novel assays with mobile phones at their core [22–25]. The availability of mobile phones, technical literacy, and remote data transmission have enabled novel methods for affecting change in Indian healthcare [26, 27] and represent a bridge to remote telemedicine. We have recently demonstrated feasibility of visual oral cancer screening using mobile phone by primary care dentists [28].

**Fig 1:**
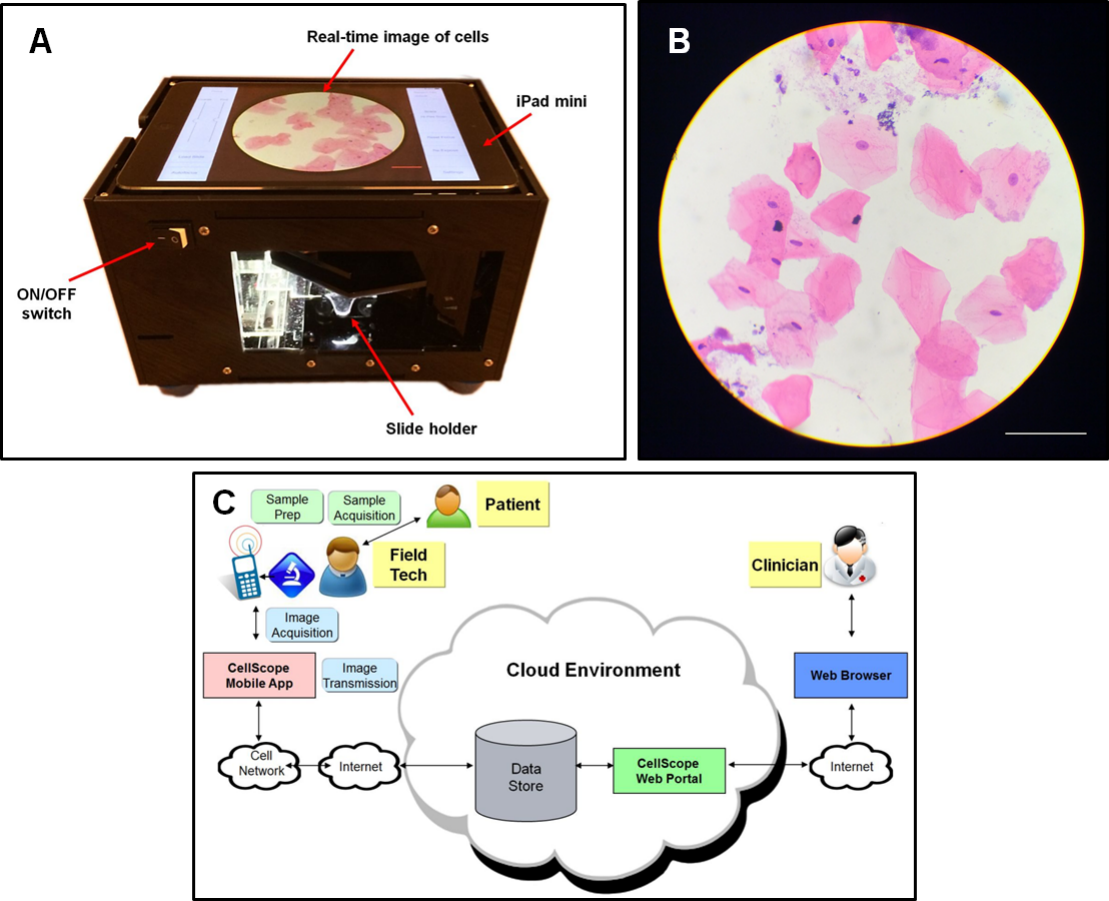
Overview of the automated CellScope device and system for mobile screening. (A) An automated image acquisition system capable of auto-focusing and scanning of a sample slide. (B) Representative image of H&E stained cells acquired with a 20X/0.4NA objective on the automated scanning CellScope. Scale bar represents 100 μm. (C) Schematic to explain the overall workflow of the telemicroscopy process.

In this study, we evaluate a 3G-telecommunication and Wi-Fi-enabled mobile microscope for oral cancer screening. The mobile microscope in this study uses a tablet to capture magnified images and automatically scan patient samples (D’Ambrosio et al, manuscript in preparation). This device, which we refer to as the automated CellScope, bypasses the need for a technician to focus and scan the sample, as well as subjectively choose suspicious fields-of-view, by automatically collecting a series of images for upload and review. The automated CellScope builds on our previous CellScope mobile phone microscopes [25] and, combined with brush biopsy and simplified staining, was evaluated at the Mazumdar Shaw Medical Foundation in Bangalore, India as part of a telemedicine screening system (Fig 1). Here we present results that validate the imaging capabilities of the device on site in India and show concordance of telepathology review of brush biopsy images against conventional cytology and histology.

## Materials and methods

### Tablet-based microscope for remote diagnosis

The automated CellScope device uses an iPad Mini to automatically collect a series of patient sample images (D’Ambrosio et al, manuscript in preparation). Briefly, a custom iOS application written for the iPad Mini 2 (Apple, Cupertino, CA) communicates with an Arduino microcontroller (Arduino Mega, Sparkfun, Boulder, CO) via Bluetooth to allow real-time gesture-based control of the imaging area (Fig 1A and 1B). The application also triggers an automated raster scan acquisition of an area of the prepared slide with automatic re-focusing on the sample during the scan. The overall workflow of the telemicroscopy process is explained in the schematic shown in Fig 1C.

The following were the main hardware components of the CellScope device:

- iPad mini 2 with 16GB storage, iOS 9.2 with Wi-Fi and 3G connectivity
- Objective lens (20x, Edmund Optics, USA) arranged such that light passing through it was directed to the iPad camera
- White LED for illumination (Rebel White LED, 700mA current draw, LuxeonStar, USA)
- A Bluetooth / Arduino-controlled, automated as well as manual gesture-based microscope stage movement
- Battery for power

### Sample collection and preparation

#### Brush biopsy

Brush biopsy was carried out using the Cytobrush Plus GT (Medesign I.C. GmbH, Germany). The liquid cytology preparation of the brush biopsy samples was performed according to the following protocol. Brush biopsy samples were immersed in 1.0 mL of SurePath cell preservative (GYN-0001-V, BD SurePath, BD Biosciences, CA, USA), and cells were released from the brush by trituration of the brush in the liquid. The cell suspension was centrifuged in a miniature centrifuge (MiniSpin, Eppendorf, Hamburg, Germany) and the supernatant was removed leaving approximately 50 μL of volume. The cells were re-suspended, placed on a slide, and then spread using the flat edge of another slide. The sample was roughly constrained to the central region of the slide to improve cell density and maximize the number of cells imaged during an automated scan of a portion of the slide area. After drying, the cells were fixed and stained as detailed below. After covering the sample with a coverslip, the prepared slide was loaded into the automated mobile microscope using a touch interface provided by a mobile app running on the iPad for image acquisition. After the images were collected, they were transmitted from the CellScope device to a web server, where pathologists performed blind review of the resulting images. Two pathologists independently viewed the digital images, cytology slides and histology slides to give their diagnoses.

#### Non-toxic H&E staining

We developed a non-toxic and environmentally non-hazardous method of staining, which in brief is as follows. Hematoxylin stock solution was prepared (1 g hematoxylin in 1 L water, 0.2 g sodium iodate, 50 g potassium aluminium sulphate dodecahydrate). The working solution was made by adding 0.1g citric acid to 100 ml stock solution. Eosin solution was made by dissolving the following in 100ml of distilled water: 0.3% (g/v) eosin G, 13% (v/v) glycerine, 0.1% (g/v) Na_2_SO_4_. After fixing cells on slide using 95% ethanol, staining was carried out by dipping the slide in hematoxylin and eosin each for 1 minute, with a wash after each stain in tap water.

### Mobile app and data acquisition

**Fig 2:**
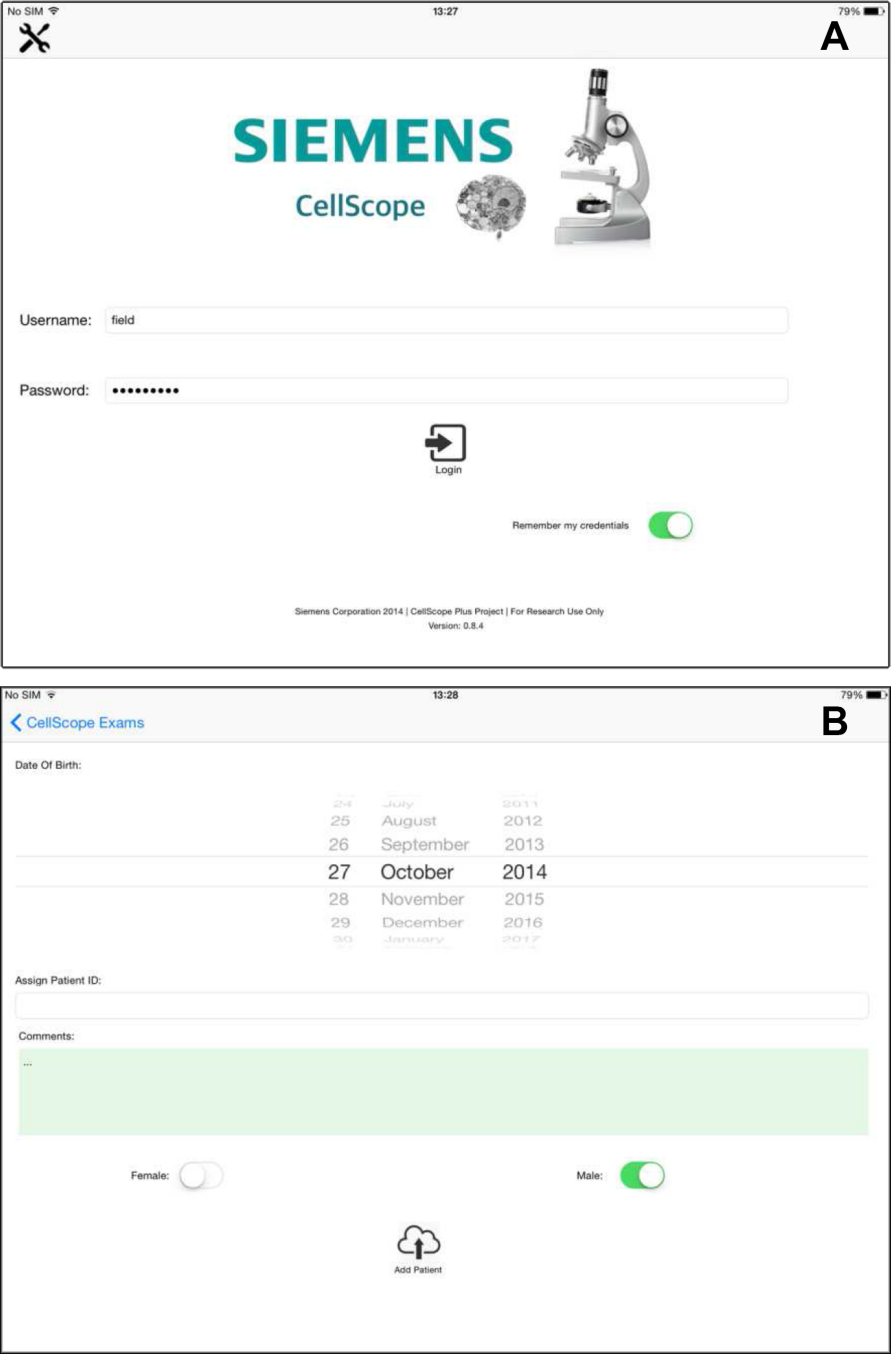
Screenshots of the iPad mobile app user interface. (A) The login page on the iPad app showing the password protected secure system for the technician. (B) Another screenshot on the iPad app showing options to enter patient date of birth, sample ID, gender and additional comments.

An app was developed for the iPad that enabled entering of patient data including case ID, age, sex and site of the lesion (Fig 2). The app also enabled Bluetooth-controlled movement of the stage, capturing images from slides, review of captured images, storage of data in the iPad and transmission of data / communication with the web server. The app provided the user the option of either autofocusing or manually adjusting the course and fine focus for image acquisition.

The mobile app was programmed to make the device automatically capture 125 images from different regions of the slide along a raster scan pattern. Images that were perceived to be blurry or that contained a cell density of less than approximately 30% (where cell density was roughly calculated as the proportion of the total field of view that was covered by cellular material) were considered inadequate and were discarded (as described in a previous study [29]). Furthermore, images which contained overlapping/clumping of cells were discarded by the technician to avoid potential issues with diagnostic interpretation. Images with a density of cells >50% were preferentially used. In some cases where the cells were considered sparse (<50% cell density), the technician took images manually using the app by panning around the slide using the touch interface and selecting fields of view with higher density of cells. A consensus of 60–100 images with high density cells was arrived at for the diagnosis following initial discussions with the two pathologists after assessment of the images of first five subjects (not included in this manuscript). The precise number of images to be captured for each slide was dictated by the technician but was within the consensus range. Following acquisition, the technician uploaded the images to the web server in bulk using the mobile app. Although not leveraged in this study, the mobile app could also be used to rapidly check the status of samples sent to the web server in real time (i.e. color-coded status results, described in the Web server section below). This functionality could be very useful in future deployment scenarios of the CellScope system (e.g. to aid remote field technicians working on rural health applications).

### Web server

**Fig 3:**
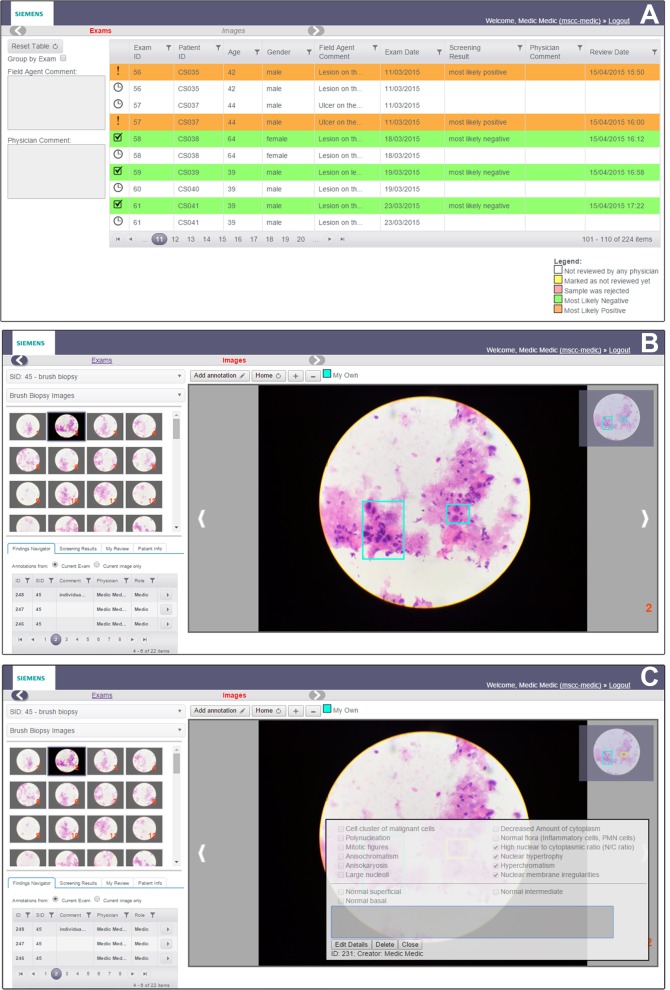
Screenshots of the CellScope server user interface. (A) Screenshot of the CellScope server (web portal interface) showing the list of patients whose diagnosis has been carried out. Color-coding of records was used to indicate the status of the samples (i.e. diagnostic result provided, awaiting review, sample rejected, etc.) (B) Screenshot of server interface showing CellScope images acquired from a patient sample. Note the thumbnail panel on the left showing thumbnails of all images captured for that patient sample and a magnified image of the selected image in the center/right. Selected regions of interest (indicated by light blue rectangles which could be drawn by the pathologists using tools provided in the interface) are overlaid on the image. (C) Screenshot of the server interface showing a pop-up window containing various cellular features which could be used by the pathologist to annotate the selected region of interest. A free-text box in this window allowed the pathologists to enter additional comments, if necessary.

A web server was developed to store images uploaded by the CellScope device and enable remote review of these images and associated data by pathologists (Fig 3). The server enabled multiple users to access it securely, each using their own login and password. The server employed a queuing system with color-coded case status (Fig 3A). The queue (list of samples in rows) represents the order in which patient samples were uploaded to the server. The status of the sample review by the pathologists was automatically pushed (i.e. status update data transmitted) from the web server to the mobile app in real time, as changes were made on the server. During observation of images, the pathologist could select and annotate any cell or group of cells by simply dragging the mouse cursor across the region of interest, resulting in a box being overlaid at that position on the microscopic image (Fig 3B). A pop-up menu of morphological features enabled easy selection of all relevant features within the selected region of interest (Fig 3C). Multiple cell selections and annotations for each patient sample were possible. Additional features such as image zooming in/out of images were also available. The pathologists entered their diagnosis of each case in the provided space. The annotations or opinions given by the pathologists were not visible to each other.

### Determination of imaging parameters for cytology

Imaging resolution and field of view can be varied by utilizing a range of objectives with mobile microscopes, as previously shown [25]. The appropriate magnification and field of view for oral cancer screening applications was decided by the technicians and pathologists involved in this study by qualitative assessment of samples imaged with the instrument. A 20X/0.4NA objective (Edmund Optics, USA) was selected to maximize field of view while providing sufficient magnification/resolution capture relevant features for diagnosis.

### Study population

The study was carried out among patients attending the dental out-patient departments of the Mazumdar-Shaw Medical Center (MSMC), Bangalore and its collaborative partner the KLE Society’s Dental College (KLESDC), Bangalore. IRB approvals for the reported studies were obtained from the respective institutions (Narayana Health Medical Ethics Committee [NHH/MEC-CL-2014/222] and Communication of Decision of the Institutional Ethics Committee, 20/11/2014). A patient consent form was prepared in four languages (English, Kannada, Hindi and Tamil). The protocol, merits and confidentiality of the study was explained to all subjects in their native language. The consent form was provided to the patient in their respective language, and was signed by them, their physician and the study investigator. One copy of the consent form was provided to each patient and one copy was kept in the MSMC library in Bangalore. Because of ethical considerations for the removal of tissue by punch biopsy from asymptomatic individuals, only patients with suspicious oral mucosal lesions were included in the study. Patients with lesions that clinically resembled obvious bullous lesions or lichen planus were excluded from sampling. Data from patients who were later found to have lesions with non-squamous pathology (sarcoma, lipoma, salivary gland tumor, bone tumor, or lymphoma) were also excluded from the study.

### Design of the study

**Fig 4:**
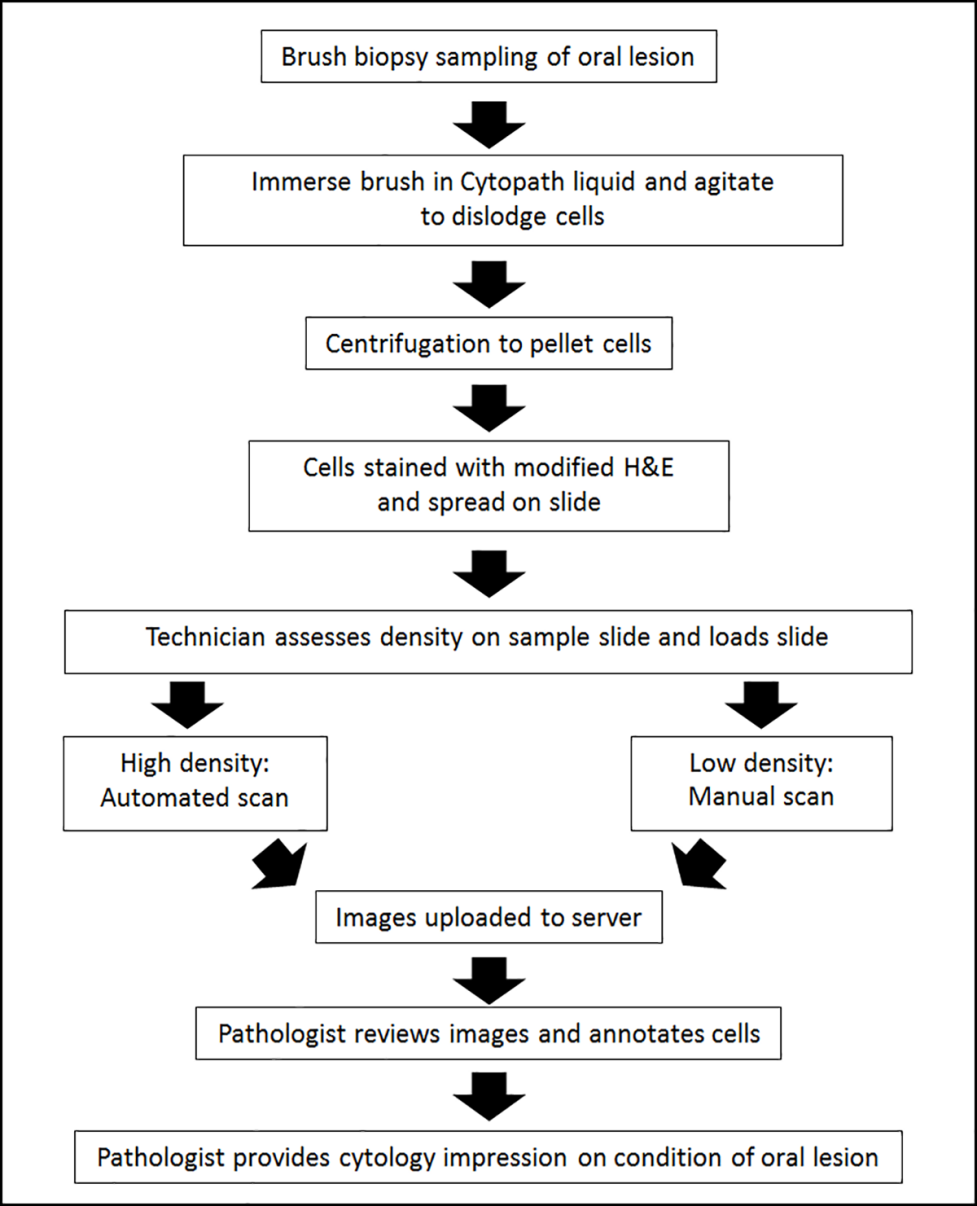
Workflow of sample acquisition and analysis for the study.

The study was designed to determine the specificity and sensitivity of a liquid cytology preparation of brush biopsy acquired samples paired with automated image acquisition. The sample and patient workflow is illustrated in Fig 4.

At first, the patients were examined by a dental surgeon. Those subjects who were identified to have clinically suspicious lesions were interrogated by liquid cytology and transfer of data carried out using the automated mobile microscope. Patients also underwent scalpel biopsy. Histological processing and examination were performed at MSMF according to standard methods. The liquid cytology preparation of the brush biopsy samples and staining was carried out as detailed above.

### Definition of positive and negative diagnosis in cytology

In this study, a patient was designated as ‘most likely positive’ if cells harvested from a patient showed features classical of atypical cells including polynucleation, mitotic figures, anisochromatism, anisokaryosis, large nucleoli, decreased amount of cytoplasm, high nucleus to cytoplasm ratio, nuclear hypertrophy, hyperchromatism, or nuclear membrane irregularities. If the cells did not show any such features, the patient was designated as ‘most likely negative’. If the slides could not be interpreted because of errors in staining, clumping of cells or poor image quality it was labelled as ‘uninterpretable’. If a lesion was later diagnosed by histology as non-squamous cell neoplasms, the patient was considered as negative, since squamous cell carcinoma was the main focus of the screening.

### Evaluation of samples by pathologists

In this proof of concept study with the CellScope telemicroscopy platform, we used two experts for comparing diagnosis. To provide a robust analysis, the following steps were taken. Two pathologists independently viewed the samples (both using a conventional microscope and digital images from the CellScope) to address the issue of ‘inter-observer variability’. The pathologists logged into the Web-based system where the CellScope images were stored and accessed the images. After looking through the images, the pathologists recorded their diagnoses. The stained slides were then assessed using conventional microscopy in a similarly blinded manner.

### Patient confidentiality and data security

Several processes were implemented to ensure patient confidentiality and data security. This included collection and handling of physical patient samples, digital transmission, and storage and analysis of CellScope images and associated patient information.

**Fig 5:**
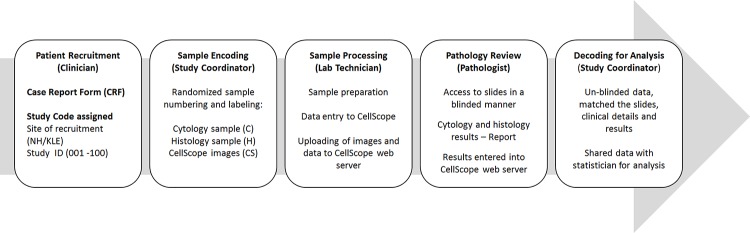
Process for encoding/decoding of patient information and samples.

The process used for encoding/decoding of all related patient information, physical samples and CellScope images to protect patient confidentiality (and also to blind diagnostic results between pathologists) is depicted in Fig 5. Clinicians collected samples from the patients and assigned an institutional ID and unique study ID to each sample, which was recorded in the Case Report Form (CRF). The samples and associated CRFs were provided to the Study Coordinator, who in turn generated a new (encoded) ID for each sample. These IDs and associated patient information was stored in a password-protected Microsoft Excel datasheet. Samples were labeled with these IDs and provided to the Lab Technician along with a specific alphanumeric code to designate the type of sample (cytology, histology, CellScope image). Some basic information associated with each sample was also provided to the Lab Technician (i.e. patient birthdate, sex, sample ID/alphanumeric code and location within the oral cavity where the sample was acquired). The Lab Technician prepared the slides, entered the basic patient data into the CellScope device and uploaded the associated images and data to the CellScope web server. Pathologists had restricted access to the web server only to analyze the sample images and enter diagnostic results in a blinded manner. Once the diagnostic reporting by the pathologists was completed, the Study Coordinator accessed the data in the web server and decoded/un-blinded the data, matching the slides, clinical details and results. All of this data (without identifying patient information) was then provided to a statistician for statistical analysis.

**Fig 6:**
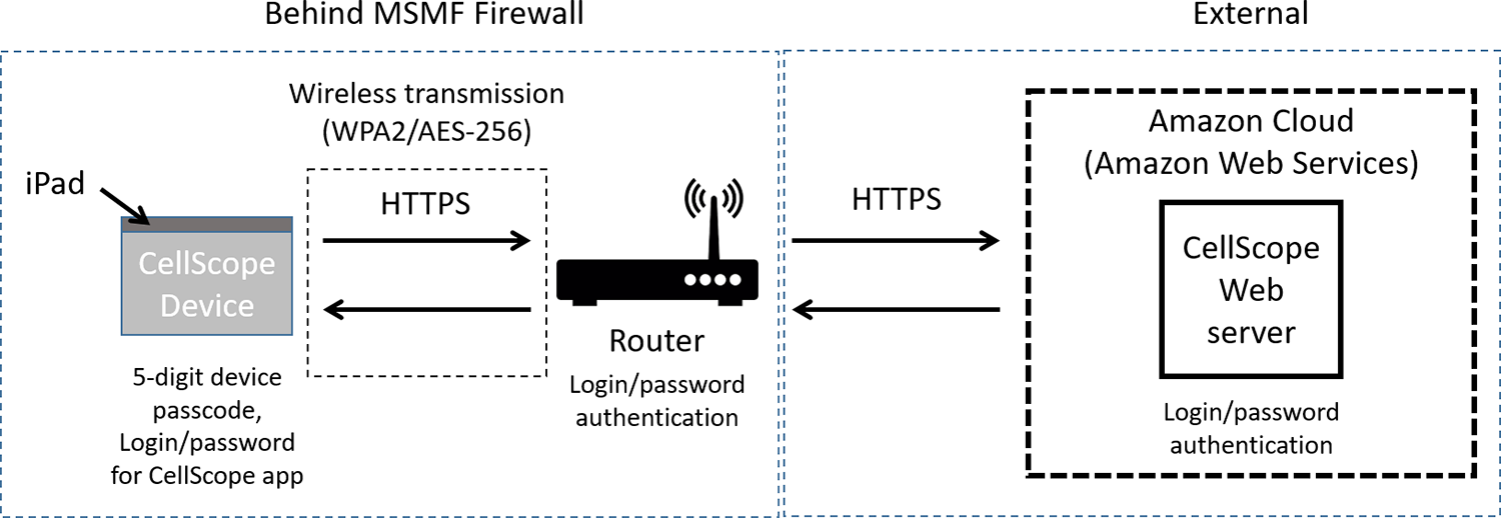
Data security for the CellScope system.

With respect to security of the CellScope device and associated data transmission, the following steps were performed. The iPad used in the CellScope device to store and transmit data was secured by a 5-digit passcode. It was restricted to use in this study and was kept physically secure at MSMF. Access to the CellScope app running on the iPad (used to control the CellScope device hardware and to transmit images) was also controlled by login/password. Data from CellScope app on the iPad was sent wirelessly (over Wi-Fi) to a local router at MSMF and from there to the CellScope web server that was hosted in the Amazon cloud (using Amazon Web Services, AWS). The router was behind the institutional firewall (not accessible from the outside internet), secured using a login and password and kept physically secure. Wireless data transmission from the iPad to the router was secured using the WPA2 authentication protocol and AES 256 bit encryption standards [30]. All data transmission between the iPad, router and AWS cloud was secured using HTTPS (Hypertext Transfer Protocol within a connection encrypted by Transport Layer Security), which provides website authentication and bidirectional encryption [31]. Amazon provides several levels of security (both physical and data-focused) for software and data that is hosted using their AWS cloud services platform (see https://aws.amazon.com/ for further information). Access to the AWS-hosted CellScope web server was controlled by a login and password system and user access logs for the server were available. The overall process for data transmission used in this study is depicted in Fig 6.

## Results

### Patient demographics

Patients recruited for the study were largely from southern states of India, including Karnataka, Tamil Nadu, Andhra Pradesh and Kerala. A total of 32 patients were evaluated in the pilot study according to the workflow in Fig 4.

A description of the basic clinical parameters of the study is provided in Tables 1 and 2. Eight of 32 patients (25%) were female. The ages of the patients ranged from 23 to 72 years (mean 50.6, S.D. 13). Twenty-five patients (78%) reported at least one of the following behaviors: consuming alcohol, chewing tobacco, smoking. Samples were collected from each patient and prepared as described above. All images were captured automatically using the CellScope device or manually by the technicians.

**Table 1:** Patient demographics and related clinical parameters of the study.

Sex			Age (28–72 years)			Risk Habits			Histopathology diagnosis		
	n	%		n	%		n	%		n	%
Male	8	25	>40	25	78	None	7	22	OSCC^a^	19	60
Female	24	75	<40	7	22	Tobacco	21	65	Lymphoma	1	3
						Tobacco and alcohol drinking	4	13	Severe dysplasia	2	6
									Moderate dysplasia	7	22
									Mild dysplasia	2	6
									Non dysplasia	1	3
Total	32	100		32	100		32	100		32	100

^a^Oral squamous cell carcinoma

**Table 2:** Clinical and pathological diagnosis of patients.

Slide #	CellScope ID	Age	Sex	Diagnosis (Dx)	Site	Histopath. Dx	Cytology Dx	CellScope Dx Pathol. 1	CellScope Dx Pathol. 2
1	CS016	42	F	Carcinoma	buccal mucosa	Positive	Positive	Negative	Positive
2	CS018	51	M	Carcinoma	tongue	Positive	Positive	Positive	Positive
3	CS003	42	M	Carcinoma	retromolar trigone	Positive	Positive	Negative	Negative
4	CS010	50	M	Carcinoma	alveolus	Positive	Positive	Negative	Positive
5	CS012	65	F	Carcinoma	tongue	Positive	Positive	Negative	Positive
6	CS014	36	M	Carcinoma	tongue	Positive	Positive	Positive	Positive
7	CS013	62	F	Carcinoma	buccal mucosa	Positive	Positive	Positive	Positive
8	CS021	72	M	Carcinoma	buccal mucosa	Positive	Positive	Positive	Positive
9	CS031	35	M	Carcinoma	buccal mucosa	Positive	Positive	Positive	Positive
10	CS032	62	M	Carcinoma	buccal mucosa	Positive	Positive	Positive	Positive
11	CS033	49	M	Carcinoma	buccal mucosa	Positive	Positive	Positive	Positive
12	CS034	53	M	Carcinoma	buccal mucosa	Positive	Positive	Positive	Positive
13	CS035	47	M	Carcinoma	tongue	Positive	Positive	Positive	Positive
14	CS037	44	F	Carcinoma	buccal mucosa	Positive	Positive	Positive	Positive
15	CS041	34	M	Carcinoma	tongue	Positive	Negative	Negative	Negative
16	CS044	67	M	Carcinoma	tongue	Positive	Positive	Positive	Positive
17	CS045	60	F	Carcinoma	labial mucosa	Positive	Positive	Positive	Positive
18	CS051	62	M	Carcinoma	tongue	Positive	Positive	Positive	Positive
19	CS002	44	M	Carcinoma	buccal mucosa	Negative	Negative	Negative	Negative
20	CS020	41	M	Tobacco pouch keratosis	buccal mucosa	Positive	Negative	Negative	Negative
21	CS022	52	M	Erythroplakia	buccal mucosa	Positive	Negative	Negative	Negative
22	CS001	61	M	Proliferative Veruccous Leukoplakia	buccal mucosa	Positive	Positive	Negative	Negative
23	CS005	23	M	Homogenous leukoplakia	buccal mucosa	Positive	Negative	Negative	Negative
24	CS008	48	M	Homogenous leukoplakia	buccal mucosa	Positive	Negative	Negative	Negative
25	CS011	65	M	Homogenous leukoplakia	buccal mucosa	Positive	Positive	Negative	Positive
26	CS019	37	M	Homogenous leukoplakia	labial mucosa	Positive	Negative	Negative	Negative
27	CS023	61	F	Reticular Lichen Planus	buccal mucosa	Positive	Negative	Negative	Negative
28	CS036	60	M	Tobacco pouch keratosis	buccal mucosa	Negative	Negative	Negative	Negative
29	CS038	64	F	Veruccous leukoplakia	buccal mucosa	Positive	Positive	Negative	Positive
30	CS039	38	M	Homogenous leukoplakia	buccal mucosa	Positive	Negative	Negative	Negative
31	CS042	65	F	Verroucous leukoplakia	buccal mucosa	Positive	Positive	Positive	Positive
32	CS043	28	M	Tobacco pouch keratosis	buccal mucosa	Positive	Negative	Negative	Negative

### Statistical analysis

For statistical analysis, the efficacy of the system (sensitivity, specificity and agreement of individual pathologists) was calculated. The specificity and sensitivity of CellScope (automated mobile microscopy) and cytology were calculated considering cytology and histology as the gold standard. All statistical analyses were done using the online calculator (http://graph pad.com). The agreement between CellScope and cytology diagnosis was examined using Kappa statistics (as described in the Results section).

### Demonstration of image capture with the automated mobile microscope

**Fig 7:**
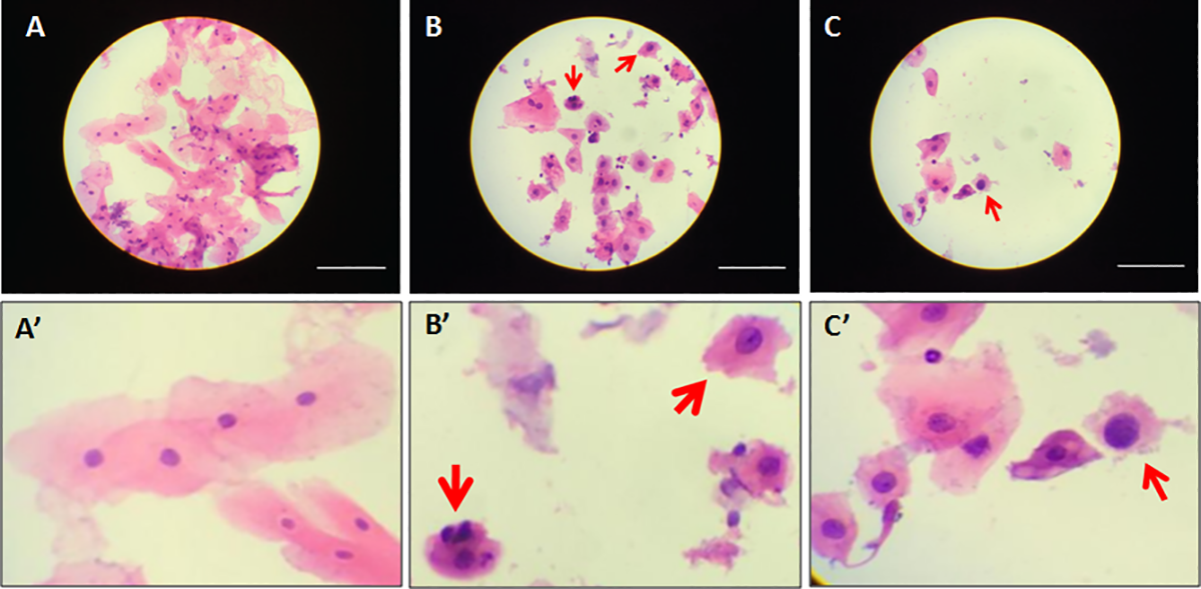
The automated mobile microscope is capable of capturing images representative of relevant disease features. **(A)** Morphologically normal cells from a brush biopsy preparation. (A’) Zoom-in of cells in A showing normal morphology. (B) Abnormal cells from a patient with confirmed malignancy, including cells with high nucleus to cytoplasm ratio and hyperchromatic nucleus indicated with red arrows. (B’) Zoom-in of suspicious cells in B showing morphological changes. (C) Suspicious cells imaged from a patient with confirmed leukoplakia. The red arrow indicates a high nucleus: cytoplasm ratio indicating a suspicious cell. Note the paucity of cells leading to difficulty with the auto-focusing algorithm for this sample preparation. (C’) Zoom-in of suspicious cell in C showing high nucleus-to-cytoplasm ratio. All scale bars represent 100 μm.

Automatic focusing takes place by taking several images at various Z-axis (focal plane) positions and determining which image has the highest contrast as measured by a Laplacian transform of the image. The successful automatic focusing and acquisition process are demonstrated for patients with a normal epithelium and those with invasive cancer (Fig 7A, 7A’, 7B and 7B’). In those patients who have leukoplakia characterized by stiffening of the epithelial surface, brush biopsy is unable to yield enough number of cells, thus causing a paucity of cells on the slide. In such cases, the technician manually selects random fields with at least some cells in them and captures images (Fig 7C and 7C’).

### Wireless data transfer

While the iPad mini had telecommunication connectivity via a 3G SIM card (which could be used for remote deployments of the CellScope device), Wi-Fi transmission was used in the clinical laboratory due to ease of use and to take advantage of faster transmission speeds during the proof-of-principle process. Duration of time taken for uploading each image using the Wi-Fi of hospital varied, depending on time of the day that this was performed. It was slowest when large number of users were using the internet and fastest before/after office hours. The time duration ranged from 6 sec to 12 sec per image. Each image was approximately 1.5 MB in size.

### Patients considered for the study

Table 2 shows the patient ages, gender, clinical diagnosis, site of sample acquisition and diagnoses by conventional histopathology and cytology. It also shows the diagnostic interpretation of the associated CellScope images provided by the two pathologists. For the histopathology results, it should be noted that samples were reported as ‘positive’ if either carcinoma or dysplasia was observed.

### Agreement between pathologists

There were two pathologists in the study, each of whom independently evaluated the cytology slides by conventional microscopy, histopathology slides by conventional microscopy and CellScope images by logging into remote server. The decision making in terms of positive/negative diagnosis by both of the pathologists was based on a set of cytological criteria. In cases where there was a difference of diagnostic opinion between the pathologists, a consensus was arrived at by reviewing each of these criteria together. Among all the cases, a discordance was observed in 5 cases and a consensus was reached for all of them after a second review. This consensus diagnosis (Table 2) for cytology or histopathology was then used to compare to the diagnosis made using automated CellScope images.

Before considering their diagnosis from evaluation of automated CellScope images, agreement between the two pathologists was calculated using the interrater reliability measure, the κ (Kappa) statistic. The κ statistic was calculated using the equation [32]:

$$\kappa =\frac{\left[\mathrm{Pr}\left(a\right)-\mathrm{Pr}\left(e\right)\right]}{\left[1-\mathrm{Pr}\left(e\right)\right]}$$

where Pr(a) represents the actual observed agreement and Pr(e) represents chance agreement. Pr(a) and Pr(e) were calculated using the online calculator available at: http://graphpad.com/quickcalcs/kappa1/. As shown in Table 3, the κ value was 0.695 for diagnosis based on automated CellScope images indicating ‘substantial’ agreement between the pathologists.

**Table 3:** Determination of kappa value between the two pathologists.

Diagnosis based on CellScope images				
		Pathologist 1		
		Yes	No	Total
**Pathologist 2**	**Yes**	14	0	14
	**No**	5	13	18
	**Total**	19	13	32
			**Pr(a)**	84.38%
			**Pr(e)**	48.83%
			**κ**	**0.695**

### Concordance of remote diagnosis from images taken by automated mobile microscopy against conventional cytology and histology

The sensitivity and specificity of the automated CellScope using either cytology or histology as Gold Standard is shown in Tables 4 and 5. These values are also provided for conventional cytology compared to histology (Table 6).

**Table 4:** Sensitivity and specificity of the automated CellScope vs. histology.

A. CellScope vs. Histology (Gold Standard)	
**Pathologist 1**	
Condition absent, test positive	0
Condition absent, test negative	2
Condition present, test positive	14
Condition present, test negative	16
**SENSITIVITY**	**47%**
**SPECIFICITY**	**100%**

**Pathologist 2**	
Condition absent, test positive	0
Condition absent, test negative	2
Condition present, test positive	19
Condition present, test negative	11
**SENSITIVITY**	**63%**
**SPECIFICITY**	**100%**

**Table 5:** Sensitivity and specificity of the automated CellScope vs. cytology.

B. CellScope vs. Cytology (Gold Standard)	
**Pathologist 1**	
Condition absent, test positive	0
Condition absent, test negative	11
Condition present, test positive	14
Condition present, test negative	7
**SENSITIVITY**	**67%**
**SPECIFICITY**	**100%**

**Pathologist 2**	
Condition absent, test positive	0
Condition absent, test negative	11
Condition present, test positive	19
Condition present, test negative	2
**SENSITIVITY**	**90%**
**SPECIFICITY**	**100%**

**Table 6:** Sensitivity and specificity of conventional cytology vs. histology.

C. Cytology vs. Histology (Gold Standard)	
Condition absent, test positive	0
Condition absent, test negative	2
Condition present, test positive	21
Condition present, test negative	9
**SENSITIVITY**	**70%**
**SPECIFICITY**	**100%**

## Discussion

Oral cancer represents a major public health challenge in India and other high-burden countries. Behavioral factors that predispose individuals to developing oral cancer and limited access to cancer care are of critical concern. Both of these increase the number of cancer cases and introduce delays in diagnosis, which in turn leads to poor prognosis. Tools to aid traditional visual examination are desperately needed in the primary care setting, where most patients present with lesions. While techniques including brush biopsies have been tested in well-equipped laboratories, applying a similar strategy in these low resource settings requires new instrumentation and information infrastructure to enable diagnosis.

In this work, we adapted a device based on mobile consumer electronics with the advantages of scale and connectivity to facilitate the imaging and transmission of a simplified, minimally invasive brush biopsy. A data upload and presentation system was developed and deployed in the clinical setting to enable review and annotation of images. We evaluated a strategy that placed varying demands on the instrument user and the instrument itself, utilizing a technician with an automated scanning mobile microscope with minimal need for user intervention.

The raw digital color (Red-Green-Blue, RGB) microscopic images used in this study were sufficient to assess the morphological characteristics of collected oral cell samples. Multiple other analogous studies carried out in the diagnosis of cervical cancer and breast cancer using a tele-cytology platform used raw RGB images for evaluating morphological characteristics [33–36]. Reading and interpretation of digital images captured by the CellScope replaced evaluation by direct viewing of the slides using a conventional microscope. Therefore, normalization of staining intensity was not necessary as the CellScope images duplicated the actual images. In both cases, diagnosis was determined using mainly morphological characteristics.

Analysis of the results indicates that the agreement between pathologists when diagnosis was done using automated CellScope images was ‘substantial’ as classified by the κ statistic value of 0.695 [32]. This indicates that the CellScope images provided sufficient, diagnostic quality information to the pathologists to form their opinions so that the agreement between them was not merely based on chance.

While the observations of only two pathologists were used in this study, we believe that based on the sample review design, this was sufficient to provide a robust analysis of samples. In another analogous telecytology based study for breast cancer, the opinions of only two cytopathologists were used for the interpretation with a concordance of 66.7%-90% [33]. In this study, digital images were found to be an excellent substitute for glass slides with the images being interpreted accurately remotely and showed excellent cellular details for cytology diagnosis. In our study, there is a concordance of 84.4% (27/32) between the pathologists in diagnosis results using the CellScope system.

The specificity of diagnosis achieved using automated CellScope images as compared to histology as gold standard was 100%. This was also the case when the diagnosis was done using automated CellScope images compared to conventional cytology as gold standard (100%). This high specificity value was found irrespective of which pathologist was doing the diagnosis. Since a high specificity points to a low rate of false positives, this indicates that as a screening tool in limited resource settings, automated CellScope would be useful to cut down cost and potentially prompt referral of suspicious lesions and lower delays in diagnosis.

The sensitivity of diagnosis achieved using CellScope images as compared to conventional cytology as gold standard varied between 67% and 90% (between the two pathologists). When the diagnosis was performed using CellScope images compared to histology as the gold standard, the sensitivity varied between 47% and 63%. Interestingly, from Table 2, it is apparent that the drop in sensitivity is due to cases of leukoplakia, where it is difficult to obtain any cells by brush biopsy, since this condition renders a hardening of the superficial layers of the buccal mucosa. A lack of cells leads to difficulty in making the correct diagnosis in cytology. Another interesting fact is that the sensitivity of diagnosis made using conventional cytology when compared to histology as gold standard is 70%, which is similar to the sensitivity using automated CellScope vs histology as gold standard. This indicates that the quality of images, sample preparation and random image selection from automated CellScope is on par with conventional cytology.

For cancer screening, a test protocol that has a high sensitivity is recommended. In a screening scenario, if the pathologist diagnoses a case as atypia, the subject is generally referred to a regional cancer center for further diagnostic confirmation. In this experimental study, screening was based on cytology and accordingly, the overall concordance was high (90%) between automated mobile microscopy and cytology when all patients were assessed together. When patient cohorts with oral cancer and dysplasia were analyzed independently, the sensitivity increased up to 94% (17/18) when comparing CellScope vs cytology. In comparison to oral cancer diagnosis, dysplasia was detected with a sensitivity of only 67% when cytology was the gold standard. These analyses gave the result that the sensitivity was high for detection of oral cancer but low for dysplasia. The low sensitivity in detection of dysplasia reduced the overall sensitivity/specificity of the study. This huge difference between diagnosing cancer and dysplasia can be accounted for by the inefficiency of cytology to diagnose atypia. Cytology could detect 95% of oral cancer cases but only 27% of the dysplastic cases, concluding that cytology as a technique had a lower sensitivity in diagnosing dysplasia. In addition, in the telemicroscopy-based diagnoses, there was a high concordance between pathologists (84.4%), demonstrating the efficacy of the CellScope system as a telemicroscopy-based diagnostic tool.

The performance of the CellScope device could be improved by further technical developments. For example, since automated image collection is preceded by liquid cytology and resuspension, the retained volume and smear size can be adjusted as a function of the centrifuged cell pellet size to maintain a consistent cell density on the slide. Alternatively, a simple threshold calculated on content in the field of view (i.e. excluding fields that contain too few cells) can rule out fields too far from the optimal cell density.

Other challenges to the performance of the automated CellScope are rooted in the brush biopsy sample preparation technique. While some work has indicated that a brush biopsy diagnosis has a sensitivity above 90% [21, 29], the results generated in this study with a conventionally read brush biopsy are more similar to other work [37], which report sensitivities around 70%. The reason for this drop in sensitivity becomes apparent upon taking a closer look at the results: in cases of hyperkeratotic lesions such as leukoplakia where there is a hardening of surface mucosal layers, the brush is unable to harvest malignant cells that are located in the deeper layers. Due to this inability to collect malignant cells, diagnosis by cytology suffers and thus sensitivity falls in cases of such lesions. Further, the lack of specific diagnostic criteria that distinguish atypical cells from precancerous lesions also contribute towards reduced sensitivity. However, in cases of more advanced lesions where malignant cells are located in upper mucosal layers, the brush easily harvests malignant cells, leading to excellent sensitivities. These facts point to limitations of the brush biopsy and cytological technique in oral cancer screening.

Since the recorded sensitivity and specificity of the traditionally read brush biopsy are comparable to measurements by the tablet-based mobile microscopes, we conclude that our imaging workflow does not cause any loss of image information. It has been reported that images acquired with iPhone mobile phones do not have loss of resolution [25, 38]. With the imaging performance demonstrated here, it would be valuable to further test the system with sample preparation techniques that provide more reproducible information about the sample or other related assays.

## Conclusions

In conclusion, this study demonstrates the potential of the CellScope mobile microscopy system to enable early detection and prevention of oral cancer in rural populations as part of a telemedicine-based screening system with remote evaluation of images.
